# Supplementary material for: YAP promotes the activation of NLRP3 inflammasome via blocking K27-linked polyubiquitination of NLRP3
Source: Nat Commun. 2021 May 11;12:2674. doi: 10.1038/s41467-021-22987-3 (PMC8113592; doi:10.1038/s41467-021-22987-3)

Supplementary Information for

# YAP promotes the activation of NLRP3 inflammasome via blocking K27-linked polyubiquitination of NLRP3

Dan Wang[1,2#], Yening Zhang[1#], Xueming Xu[1], Jianfeng Wu[3], Yue Peng[4], Jing Li[1], Ruiheng Luo[1], Lingmin Huang[1], Liping Liu[5], Songlin Yu[1,6], Ningjie Zhang[7], Ben Lu[1,8,9]*, Kai Zhao[1,*]

[1]Department of Hematology and Key Laboratory of Non-resolving Inflammation and Cancer of Hunan Province, the Third Xiangya Hospital, Central South University, Changsha, Hunan Province, 410000 P. R. China
[2]Department of Dermatology, The Third Xiangya Hospital, Central South University, Changsha, Hunan Province, 410000 P. R. China
[3]State Key Laboratory of Cellular Stress Biology Innovation Center for Cell Signaling Network, School of Life Sciences, Xiamen University, Xiamen, Fujian Province 361005, P.R. China.
[4]Department of Critical Care Medicine, The Third Xiangya Hospital, Central South University, Changsha, Hunan Province, 410000 P. R. China
[5]Department of General Surgery, The Third Xiangya Hospital, Central South University, Changsha, Hunan Province, 410000 P. R. China
[6]Postdoctoral Research Station of Clinical Medicine, The Third Xiangya Hospital, Central South University, Changsha, Hunan Province, 410000 P. R. China
[7]Department of Blood Transfusion, The Second Xiangya Hospital, Central South University, Changsha, Hunan Province, 410000 P. R. China
[8]Department of Pathophysiology, School of Basic Medical Science, Central South University, Changsha, Hunan Province, 410000 P. R. China
[9]Key Laboratory of sepsis and translational medicine, School of Basic Medical Science, Central South University, Changsha, Hunan Province, 410000 P.R. China

[#] These authors contributed equally to this work.
*Correspondence: xybenlu@csu.edu.cn; kaizhao@csu.edu.cn

**Supplementary Figure 1. YAP promotes non-canonical inflammasome activation**

**a** Immunoblot or Q-PCR analysis of protein (top) and mRNA (bottom) expression of YAP in mouse peritoneal macrophages 48hr after transfection with control siRNA (Ctrl siRNA) or siRNA specific for YAP (YAP siRNA). Data were normalized to the expression of β-actin reference (mean ± SD, unpaired Two-tailed t test, YAP siRNA vs. Ctrl siRNA lower panel: ***P < 0.0001; n=3 independent experiments). **b** ELISA of IL-1β in supernatants of mouse peritoneal macrophages from *Yap$^{fl/fl}$ lyz2-Cre* or *Yap$^{fl/fl}$* mice, treated with indicated stimuli (mean ± SD, Two-Way ANOVA with Bonferroni test, *Yap$^{fl/fl}$ lyz2-Cre* vs. *Yap$^{fl/fl}$*, left panel: ***P=0.0002, **P=0.0011 in sequence; n=3 independent experiments). **c** Immunoblot analysis of supernatants (SN) or cell lysates (CL) of mouse peritoneal macrophages from *Yap$^{fl/fl}$ lyz2-Cre* or *Yap$^{fl/fl}$* mice, treated with indicated stimuli. Similar results were obtained from three independent experiments. Source data are provided as a Source Data file.

**Supplementary Figure 2. YAP localized in the cytoplasm of macrophages upon LPS stimulation**

**a** Q-PCR analysis of NLRP3 and IL-1β mRNA expression in *Yap$^{fl/fl}$ lyz2-Cre* or *Yap$^{fl/fl}$* mouse peritoneal macrophages stimulated for indicated times with LPS (mean ± SD, Two-Way ANOVA with Bonferroni test, n=3 independent experiments). **b** HEK293T cells transfected with Myc-YAP WT or Myc-YAP S112A were fixed, incubated with anti-Myc antibody and subsequently a secondary antibody conjugated to DyLight 488. YAP WT or YAP S112A distribution was examined by Confocal microscopy, Scale bars, 10 μm. **c** Immunoblot analysis of cytoplasmic or nuclear protein from mouse peritoneal macrophages stimulated for indicated times with LPS. **d** Mouse peritoneal macrophages immunofluorescence of YAP followed stimulating for indicated times with LPS. Image visualization was made on Confocal microscopy, Scale bars, 10 μm. Similar results were obtained from three independent experiments. Source data are provided as a Source Data file.

**Supplementary Figure 3. Hippo pathway is activated by serum starvation or high**

**cell confluence**

**a,b** Immunoblot analysis of cell lysates from mouse peritoneal macrophages treated by serum starvation for indicated times (**a**) or seeded into different confluence (**b**). **c** Q-PCR analysis of Lats1 or Lats2 mRNA expression in mouse peritoneal macrophages 48h after transfection with control siRNA (si-Ctrl) or siRNA specific for LATS1/2 (si-Lats1/2) (mean ± SD, Two-Way ANOVA with Bonferroni test, Lats1 siRNA vs. Ctrl siRNA: ***P < 0.0001; Lats1 siRNA vs. Ctrl siRNA: ***P < 0.0001; n=3 independent experiments). **d** Immunoblot analysis of cell lysates from mouse peritoneal macrophages silenced of Lats1/2 and treated with serum starvation for indicated times. **e** Immunoblot of TAZ protein level in mouse peritoneal macrophages stimulating with LPS for indicated times and positive control extracts from lung tissues. **f** Q-PCR analysis of Taz mRNA expression in mouse peritoneal macrophages stimulating with LPS for indicated times and positive control extracts from lung tissues (mean ± SD, One-Way ANOVA with Bonferroni test, Lung tissues vs macrophage treated with LPS 0h, LPS 2h, LPS 4h or LPS 8h: ***P < 0.0001, ***P < 0.0001, ***P < 0.0001, ***P < 0.0001 in sequence; n=3 independent experiments). Similar results were obtained from three independent experiments. Source data are provided as a Source Data file.

**Supplementary Figure 4. β-TrCP1 interacts with NLRP3**

**a** Immunoblot analysis of lysates from HEK293T cells transfected with HA-tagged ubiquitin (HA-Ub), Myc-NLRP3, with or without Flag-14-3-3σ, followed by IP with anti-Myc, probed with anti-HA. **b** Immunoblot analysis of lysates from HEK293T cells transfected with Myc-NLRP3 and Flag-β-TrCP1, followed by IP with anti-Myc (left) or anti-Flag (right), probed with anti-Flag (left) or anti-Myc (right). **c** Immunoblot analysis of cell lysates from mouse peritoneal macrophages stimulated with LPS for 4 h or not, followed by IP with anti-β-TrCP1, probed with anti-NLRP3. **d** Mouse peritoneal macrophages immunofluorescence of NLRP3 and β-TrCP1 after stimulated for indicated times with LPS. NLRP3 and βTrCP1 co-localization were examined by Confocal microscopy, Scale bars, 5 μm. **e** Schematic diagram of NLRP3 protein sequence and two potential phosphodegron motifs. **f** Immunoblot analysis of lysates

from HEK293T cells transfected with Flag-β-TrCP1, Myc-NLRP3 WT or Myc-NLRP3 S891A/S895A or Myc-NLRP3 S194A/S197A/S198A, followed by IP with anti-Myc, probed with anti-Flag. Similar results were obtained from three independent experiments. Source data are provided as a Source Data file.

**Supplementary Figure 5. YAP disrupts the interaction between NLRP3 and β-TrCP1 through competing with β-TrCP1 to bind NLRP3**

**a** Immunoblot analysis of lysates from iBMDMs with or without overexpressed YAP, stimulated with LPS for 4 h or not, followed by IP with anti-NLRP3, probed with anti-p-ser. **b** Immunoblot analysis of lysates from HEK293T cells transfected with Flag-β-TrCP1, His-YAP WT or His-YAP S366A, followed by IP with anti-Flag, probed with anti-His. **c** Immunoblot analysis of lysates from HEK293T cells transfected with Flag-β-TrCP1 and Myc-NLRP3, His-YAP WT or His-YAP S366A, followed by IP with anti-Flag, probed with anti-Myc. **d** Immunoblot analysis of lysates from HEK293T cells transfected with Myc-NLRP3 and Flag-YAP, followed by IP with anti-Myc (above) or anti-Flag (below), probed with anti-Flag (above) or anti-Myc (below). **e, f** The physical interaction between NLRP3 and YAP (**e**) or β-TrCP1 (**f**) were visualized as the red spots by PLA in mouse peritoneal macrophages stimulated with LPS for 4h. Scale bars, 5 μm. **g** Schematic diagram of YAP and its truncation mutants. **h** Immunoblot analysis of lysates from HEK293T cells transfected with Myc-NLRP3, and His-YAP fl or His-YAP 1-150 or His-YAP 151-488, followed by IP with anti-Myc, probed with anti-His. **i** Immunoblot analysis of lysates from HEK293T cells transfected with Myc-NLRP3 and Flag-β-TrCP1, and His-YAP fl or His-YAP 1-150 or His-YAP 151-488, followed by IP with anti-Myc, probed with anti-Flag. **j** Schematic diagram of NLRP3 and its truncation mutants. **k, l** Myc-tagged NLRP3 or its mutants(△PYD,△NACHT, and△LRR) and Flag-β-TrCP1 (**k**) or Flag-YAP (**l**) were individually transfected into HEK293T cells. The cell lysates were immunoprecipitated with an anti-Flag antibody and then immunoblotted with the indicated antibodies. Similar results were obtained from three independent experiments. Source data are provided as a Source Data file.

**Supplementary Figure 6. Identification ubiquitination site of NLRP3**

**a** Immunoblot analysis of lysates from HEK293T cells transfected with HA-tagged K27-linked ubiquitin (K27-Ub), Flag- β-TrCP1 and Myc-NLRP3, Myc-NLRP3△PYD, Myc-NLRP3 △ NACHT, or Myc-NLRP3 △ LRR , followed by IP with anti-Myc, probed with anti-HA. **b** Alignment of NLRP3 sequences of different species, highlighted is the conserved Lys. Similar results were obtained from three independent experiments. Source data are provided as a Source Data file.

**Supplementary Figure 7. Gate strategies of neutrophils in peritoneal lavage fluid**

Peritoneal lavage fluid from *Yap*$^{fl/fl}$ *lyz2-Cre* or *Yap*$^{fl/fl}$ mice injected with MSU were assessed by flow cytometry. The Gated neutrophils are CD11b$^{+}$ Ly-6G$^{+}$

**Supplementary Table 1. Sequences of PCR primers used in this study**

| Name | Prime | Sequence |
|---|---|---|
| *mYap* | Forward | 5’-CCCGACTCCTTCTTCAAGC-3’ |
| | Reverse | 5’-CTCGAACATGCTGTGGAGTC-3’ |
| *mNlrp3* | Forward | 5’-TGGATGGGTTTGCTGGGAT-3’ |
| | Reverse | 5’-CTGCGTGTAGCGACTGTTGAG-3’ |
| *mIL-1β* | Forward | 5’- GCAACTGTTCCTGAACTCAACT-3’ |
| | Reverse | 5’- ATCTTTTGGGGTCCGTCAACT-3’ |
| *mβ-Trcp1* | Forward | 5’-AAGACTGTAATAATGGCGAACCC-3’ |
| | Reverse | 5’-TCTCTTGGTTTATGCAAAGCCTG-3’ |
| *mLats1* | Forward | 5’-AAAGCCAGAAGGGTACAGACA-3’ |
| | Reverse | 5’-CCTCAGGGATTCTCGGATCTC-3’ |
| *mLats2* | Forward | 5’- GGACCCCAGGAATGAGCAG -3’ |
| | Reverse | 5’- CCCTCGTAGTTTGCACCACC -3’ |
| *mTaz* | Forward | 5’- CATGGCGGAAAAAGATCCTCC -3’ |
| | Reverse | 5’- GTCGGTCACGTCATAGGACTG -3’ |
| *mβ-actin* | Forward | 5’-AGTGTGACGTTGACATCCGT-3’ |
| | Reverse | 5’-GCAGCTCAGTAACAGTCCGC-3’ |

# Supplemental Fig.1

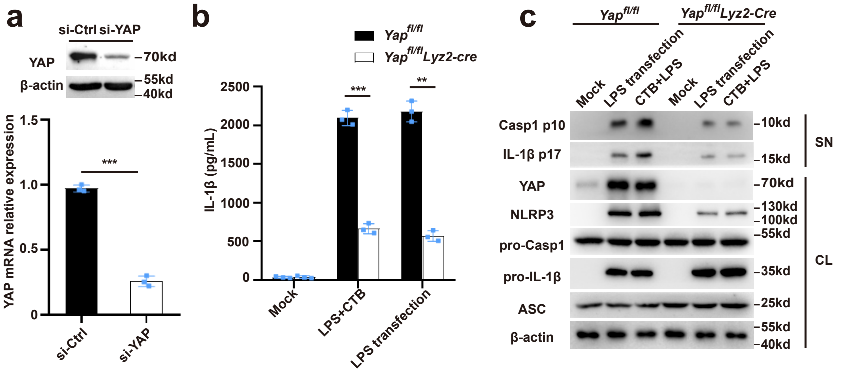

# Supplemental Fig.2

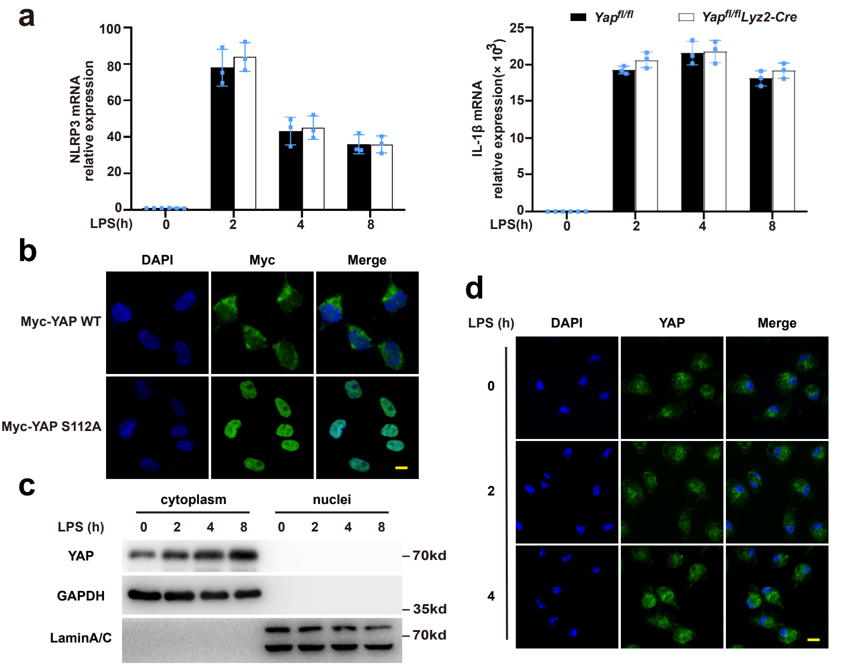

# Supplemental Fig.3

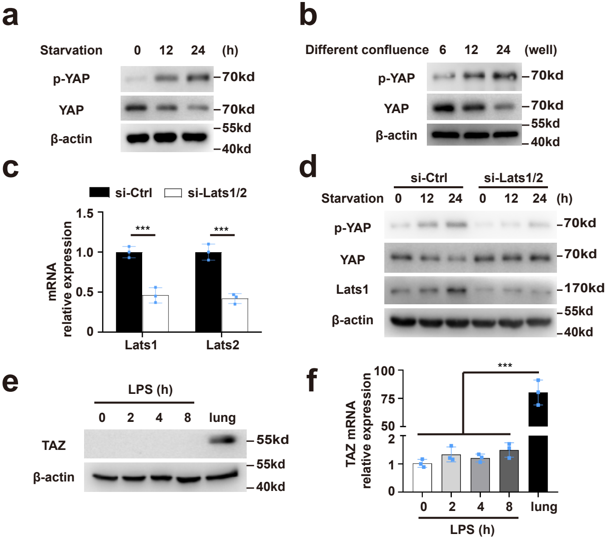

Supplemental Fig.4

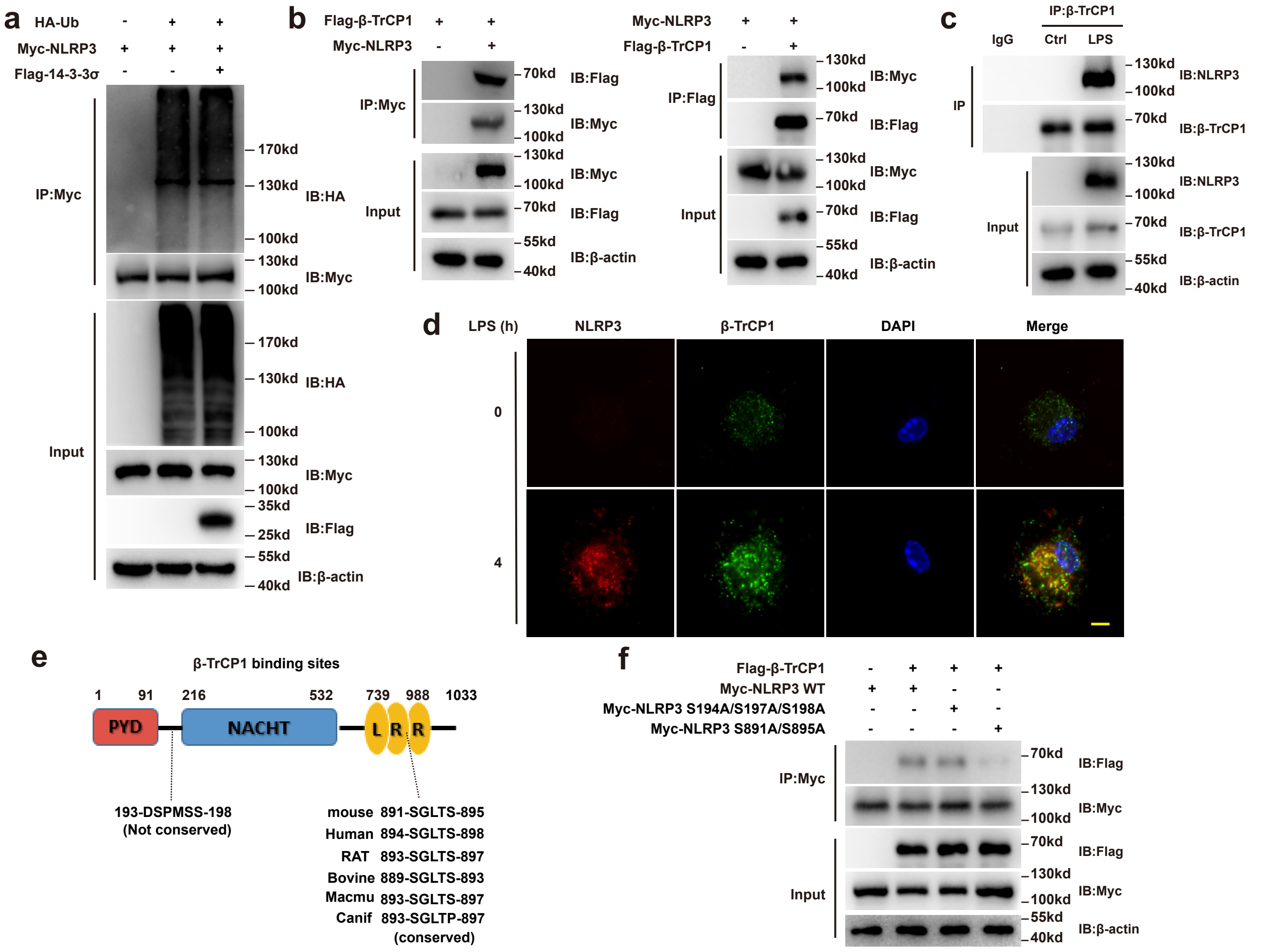

Supplemental Fig.5

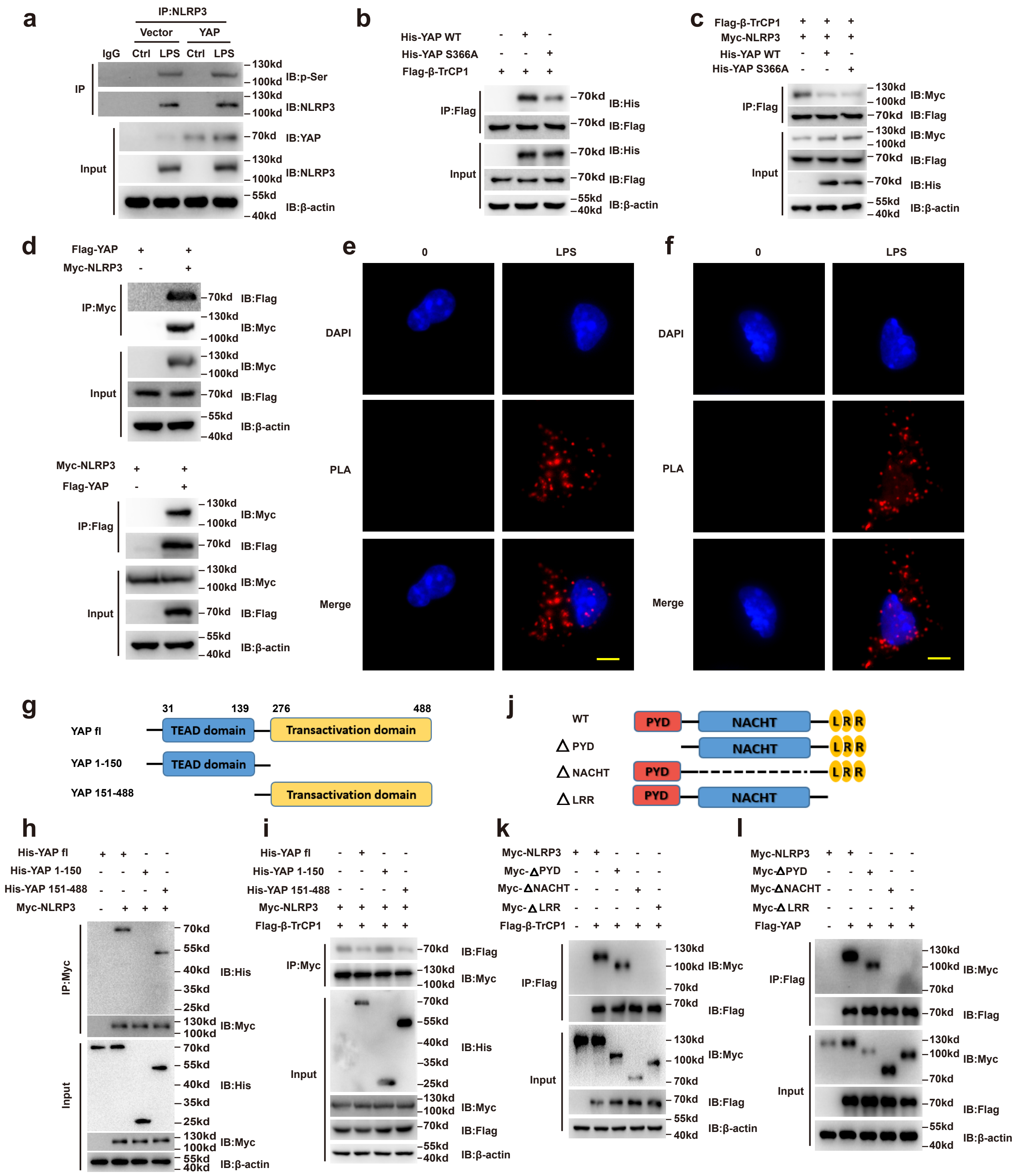

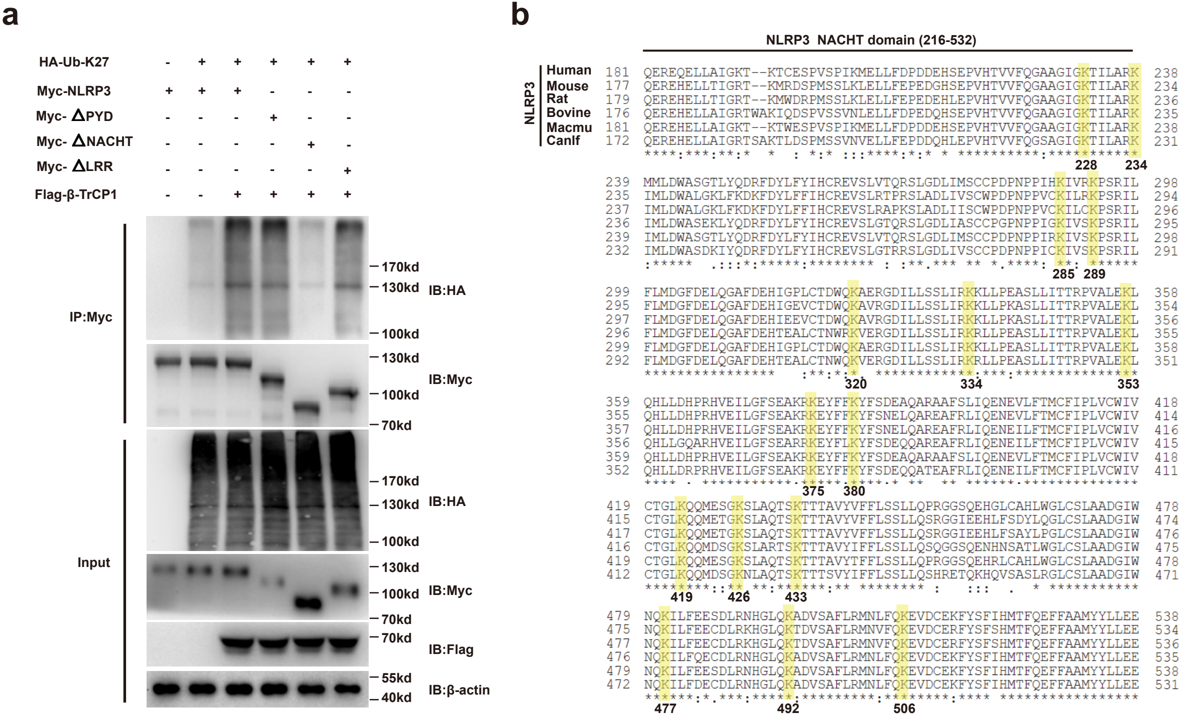
a
HA-Ub-K27 - + + + + +
Myc-NLRP3 + + + - - -
Myc- ΔPYD - - - + - -
Myc- ΔNACHT - - - - + -
Myc- ΔLRR - - - - - +
Flag-β-TrCP1 - - + + + +
IP:Myc
Input
170kd
130kd
100kd
70kd
55kd
40kd
IB:HA
IB:Myc
IB:Flag
IB:β-actin
b
NLRP3 NACHT domain (216-532)
NLRP3
Human
Mouse
Rat
Bovine
Macmu
Canlf
181 QEREQELLAIGKT--KTCESPVSPIKMELLFDPDDEHSEPVHTVVFQGAAGIGKTILARK 238
177 QEREHELLTIGRT--KMRDSPMSSLKLELLFEPEDGHSEPVHTVVFQGAAGIGKTILARK 234
179 QEREHELLTIGRT--KMWDRPMSSLKLELLFEPEDEHLEPVHTVVFQGAAGIGKTILARK 236
176 QEREHELLAIGRTWAKIQDSPVSSVNLELLFDPEDQHSEPVHTVVFQGAAGIGKTILARK 235
181 QEREHELLAIGKT--KTWESPVSPIKMELLFDPDDEHSEPVHTVVFQGAAGIGKTILARK 238
172 QEREHELLAIGRTSAKTLDSPMSSVNVELLFEPDDQHLEPVHTVVFQGSAGIGKTILARK 231
228 234
239 MMLDWASGTLYQDRFDYLFYIHCREVSLVTQRSLGDLIMSCCPDPNPPIHKIVRKPSRIL 298
235 IMLDWALGKLFKDKFDYLFFIHCREVSLRTPRSLADLIVSCWPDPNPPVCKILRKPSRIL 294
237 IMLDWALGKLFKDKFDYLFFIHCREVSLRAPKSLADLIISCWPDPNPPVCKILCKPSRIL 296
236 IMLDWASEKLYQDRFDYLFYIHCREVSLGTQRSLGDLIASCCPGPNPPIGKIVSKPSRIL 295
239 IMLDWASGTLYQDRFDYLFYIHCREVSLVTQRSLGDLIMSCCPDPNPPIRKIVSKPSRIL 298
232 IMLDWASDKIYQDRFDYLFYIHCREVSLGTRRSLGDLIVSCCPDPNPPICKIVSKPSRIL 291
285 289
299 FLMDGFDELQGAFDEHIGPLCTDWQKAERGDILLSSLIRKKLLPEASLLITTRPVALEKL 358
295 FLMDGFDELQGAFDEHIGEVCTDWQKAVRGDILLSSLIRKKLLPKASLLITTRPVALEKL 354
297 FLMDGFDELQGAFDEHIEEVCTDWQKAVRGDILLSSLIRKKLLPKASLLITTRPVALEKL 356
296 FLMDGFDELQGAFDEHTEALCTNWRKVERGDILLSSLIRKRLLPEASLLITTRPVALEKL 355
299 FLMDGFDELQGAFDEHIGPLCTDWQKAERGDILLSSLIRKKLLPEASLLITTRPVALEKL 358
292 FLMDGFDELQGAFDEHTEALCTNWQKVERGDILLSSLIRKRLLPEASLLITTRPVALEKL 351
320 334 353
359 QHLLDHPRHVEILGFSEAKRKEYFFKYFSDEAQARAAFSLIQENEVLFTMCFIPLVCWIV 418
355 QHLLDHPRHVEILGFSEAKRKEYFFKYFSNELQAREAFRLIQENEVLFTMCFIPLVCWIV 414
357 QHLLDHPRHVEILGFSEAKRKEYFFKYFSNELQAREAFRLIQENEILFTMCFIPLVCWIV 416
356 QHLLGQARHVEILGFSEARRKEYFLKYFSDEQQAREAFRLIQENEILFTMCFIPLVCWIV 415
359 QHLLDHPRHVEILGFSEAKRKEYFFKYFSDEAQARAAFSLIQENEVLFTMCFIPLVCWIV 418
352 QHLLDRPRHVEILGFSEAKRKEYFFKYFSDEQQATEAFRLIQENEILFTMCFIPLVCWIV 411
375 380
419 CTGLKQQMESGKSLAQTSKTTTAVYVFFLSSLLQPRGGSQEHGLCAHLWGLCSLAADGIW 478
415 CTGLKQQMETGKSLAQTSKTTTAVYVFFLSSLLQSRGGIEEHLFSDYLQGLCSLAADGIW 474
417 CTGLKQQMETGKSLAQTSKTTTAVYVFFLSSLLQSRGGIEEHLFSAYLPGLCSLAADGIW 476
416 CTGLKQQMDSGKSLARTSKTTTAVYIFFLSSLLQSQGGSQENHNSATLWGLCSLAADGIW 475
419 CTGLKQQMESGKSLAQTSKTTTAVYTFFLSSLLQPRGGSQEHRLCAHLWGLCSLAADGIW 478
412 CTGLKQQMDSGKNLAQTSKTTTSVYIFFLSSLLQSHRETQKHQVSASLRGLCSLAADGIW 471
419 426 433
479 NQKILFEESDLRNHGLQKADVSAFLRMNLFQKEVDCEKFYSFIHMTFQEFFAAMYYLLEE 538
475 NQKILFEECDLRKHGLQKTDVSAFLRMNVFQKEVDCERFYSFSHMTFQEFFAAMYYLLEE 534
477 NQKILFEECDLRKHGLQKTDVSAFLRMNVFQKEVDCERFYSFSHMTFQEFFAAMYYLLEE 536
476 NQKILFQECDLRNHGLQKADVSAFLRMNLFQKEVDCEKFYSFIHMTFQEFFAAMYYLLEE 535
479 NQKILFEESDLRNHGLQKADVSAFLRMNLFQKEVDCEKFYSFIHMTFQEFFAAMYYLLEE 538
472 NQKILFDECDLRNHGLQKADVSAFLRMNLFQKEVDCEKFYSFIHMTFQEFFAAMYYLLEE 531
477 492 506

Supplemental Fig.7

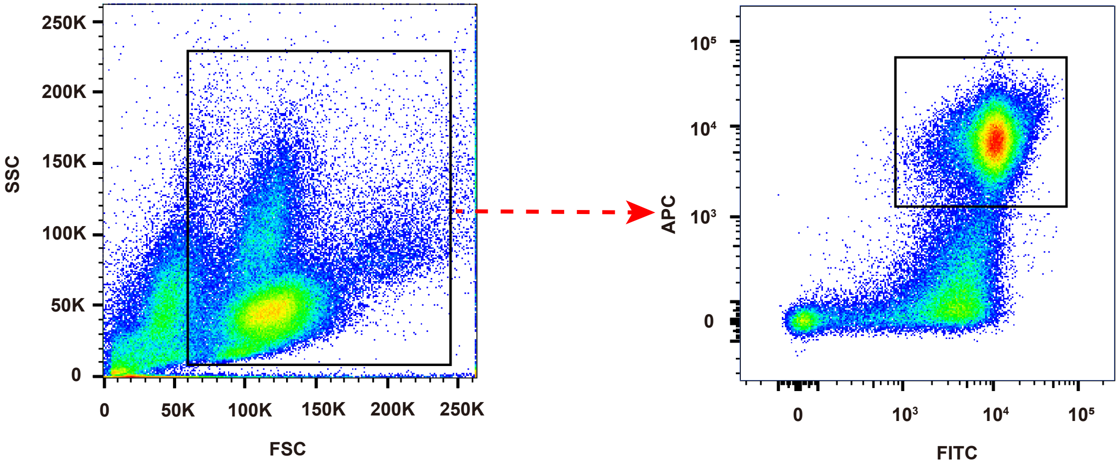

Supplement: Supplementary file 1 — Supplementary Information [file 41467_2021_22987_MOESM1_ESM.pdf]
