# Supplementary material for: YAP promotes the activation of NLRP3 inflammasome via blocking K27-linked polyubiquitination of NLRP3
Source: Nat Commun. 2021 May 11;12:2674. doi: 10.1038/s41467-021-22987-3 (PMC8113592; doi:10.1038/s41467-021-22987-3)
Supplement: Supplementary file 3 — Reporting Summary [file 41467_2021_22987_MOESM3_ESM.pdf]

nature research

Corresponding author(s): Kai Zhao

Last updated by author(s): Mar 21, 2021

Reporting Summary

Nature Research wishes to improve the reproducibility of the work that we publish. This form provides structure for consistency and transparency in reporting. For further information on Nature Research policies, see our Editorial Policies and the Editorial Policy Checklist.

Statistics

For all statistical analyses, confirm that the following items are present in the figure legend, table legend, main text, or Methods section.

n/a	Confirmed	
☐	☒	The exact sample size (*n*) for each experimental group/condition, given as a discrete number and unit of measurement
☐	☒	A statement on whether measurements were taken from distinct samples or whether the same sample was measured repeatedly
☐	☒	The statistical test(s) used AND whether they are one- or two-sided<br>*Only common tests should be described solely by name; describe more complex techniques in the Methods section.*
☐	☒	A description of all covariates tested
☐	☒	A description of any assumptions or corrections, such as tests of normality and adjustment for multiple comparisons
☐	☒	A full description of the statistical parameters including central tendency (e.g. means) or other basic estimates (e.g. regression coefficient) AND variation (e.g. standard deviation) or associated estimates of uncertainty (e.g. confidence intervals)
☐	☒	For null hypothesis testing, the test statistic (e.g. *F*, *t*, *r*) with confidence intervals, effect sizes, degrees of freedom and *P* value noted<br>*Give P values as exact values whenever suitable.*
☒	☐	For Bayesian analysis, information on the choice of priors and Markov chain Monte Carlo settings
☒	☐	For hierarchical and complex designs, identification of the appropriate level for tests and full reporting of outcomes
☒	☐	Estimates of effect sizes (e.g. Cohen's *d*, Pearson's *r*), indicating how they were calculated

Our web collection on statistics for biologists contains articles on many of the points above.

Software and code

Policy information about availability of computer code

Data collection	There is no previously unreported custom computer code or algorithm used to generate results in this manuscript.
Data analysis	Statistics analysis: GraphPad Prism 8; Image Quantification: Image J 1.52a; FlowJo 10

For manuscripts utilizing custom algorithms or software that are central to the research but not yet described in published literature, software must be made available to editors and reviewers. We strongly encourage code deposition in a community repository (e.g. GitHub). See the Nature Research guidelines for submitting code & software for further information.

Data

Policy information about availability of data

All manuscripts must include a data availability statement. This statement should provide the following information, where applicable:

- Accession codes, unique identifiers, or web links for publicly available datasets
- A list of figures that have associated raw data
- A description of any restrictions on data availability

The authors declare that the data supporting the findings of this study are available within the paper and its supplementary information files or available from the corresponding author upon reasonable request. Source data are provided with this paper.

Field-specific reporting

Life sciences study design

All studies must disclose on these points even when the disclosure is negative.

Sample size	The authors declare that the data supporting the findings of this study are available within the paper and its supplementary information files or available from the corresponding author upon reasonable request. Source data are provided with this paper.
Data exclusions	No data exclusions.
Replication	Similar results were obtained from three independent experiments. All attempts at replication were successful.
Randomization	All animals used in this manuscript were allocated into experimental groups randomly.
Blinding	A researcher blinded to the group allocation was responsible for the data collection and final data analysis.

Reporting for specific materials, systems and methods

We require information from authors about some types of materials, experimental systems and methods used in many studies. Here, indicate whether each material, system or method listed is relevant to your study. If you are not sure if a list item applies to your research, read the appropriate section before selecting a response.

Materials & experimental systems

n/a	Involved in the study	
☐	☒	Antibodies
☐	☒	Eukaryotic cell lines
☒	☐	Palaeontology and archaeology
☐	☒	Animals and other organisms
☒	☐	Human research participants
☒	☐	Clinical data
☒	☐	Dual use research of concern

Methods

n/a	Involved in the study	
☒	☐	ChIP-seq
☐	☒	Flow cytometry
☒	☐	MRI-based neuroimaging

Antibodies

Antibodies used	Anti-Caspase-1 antibody (Abcam, 1:1000, ab179515), anti-IL-1β antibody (RD systems, 1:1000, AF-401-NA; RRID: AB_416684), anti-YAP1 antibody (ABclonal, 1:1000, A1002), anti-TAZ antibody (ABclonal, 1:1000, A15806), anti-NLRP3 antibody (Adipogen, 1:1000, Cryo-2), anti-ASC antibody (Adipogen, 1:1000, AL177), anti-β-actin antibody (Cell Signaling Technology, 1:10000, BH10D10), anti-Phospho-YAP(Ser127) antibody (Cell Signaling Technology, 1:1000, 4911), anti-β-TrCP antibody (Cell Signaling Technology, 1:1000, D13F10), anti-LATS1 antibody (Affinity, 1:1000, AF7669), anti-Ub antibody (Santa cruz, 1:200, Sc-8017), anti-HA-tag (MBL, 1:5000, M180-3), anti-DDDDK-tag (MBL, 1:5000, M185-3L), anti-Myc-tag (MBL, 1:5000, M047-3), anti-His-tag (MBL, 1:5000, D291-3) were used for Western bloting. Anti-NLRP3 antibody (Adipogen, 1:400, Cryo-2) and mouse immunoglobin IgG protein (Santa cruz, 1:160, SC-2025) were used for IP. DyLight 488-labeled secondary antibody (InvivoGen, 1:50, A120-100D2) and Alexa Fluor 594-conjugated secondary antibody (InvivoGen, 1:50, 405326) were used for immunofluorescence. FITC anti-mouse/human CD11b (Biolegend, 1:500, 101216) and APC anti-mouse Ly-6G (Biolegend, 1:500,127614) were used for flow cytometry.
Validation	All antibodies were purchased from the companies described in the manuscript, and the blot sample figure and validation wereavailable on the manufacturer's website.

Eukaryotic cell lines

Policy information about cell lines

Cell line source(s)	HEK293T cells were obtained from ATCC; iBMDMs were provided by Dr. Feng Shao.
Authentication	All cell lines were authenticated by STR profiling .
Mycoplasma contamination	All cell lines were routinely verified to be free of mycoplasma contamination.
Commonly misidentified lines (See ICLAC register)	No commonly misidentified cells were used.

Animals and other organisms

Policy information about studies involving animals; ARRIVE guidelines recommended for reporting animal research

Laboratory animals	YAP fl/fl mice and Lyz2-Cre mice were from Jackson laboratories, C57BL/6 mice were from Hunan SJA Laboratory Animal Co.Ltd (Changsha, China). All mice with same gender were used between 8 and 10 weeks of age
Wild animals	The study did not involve wild animals.
Field-collected samples	The study did not involve filed-collected samples.
Ethics oversight	Mice were housed in a pathogen-free temperature-controlled environment under a 12 h light at 23 °C and a 12 h of dark at 21 °C with free access to water and standard rodent chow diet. All animal procedures were performed with an approved protocol from the Institutional Animal Care and Use Committee of Central South University.

Note that full information on the approval of the study protocol must also be provided in the manuscript.

Flow Cytometry

Plots

Confirm that:

- [x] The axis labels state the marker and fluorochrome used (e.g. CD4-FITC).
- [x] The axis scales are clearly visible. Include numbers along axes only for bottom left plot of group (a 'group' is an analysis of identical markers).
- [x] All plots are contour plots with outliers or pseudocolor plots.
- [x] A numerical value for number of cells or percentage (with statistics) is provided.

Methodology

Sample preparation	Peritoneal exudate cells were obtained and blocked with anti-CD16/32 antibody for 30min, then incubated with FITC-mouse CD11b antibody, APC-mouse Ly-6G antibody for 30 min. After washing three times with PBS, stained cells were analyzed on the BD FACSCalibur to detect the numbers of neutrophils.
Instrument	BD FACSCablibur
Software	FlowJo 10
Cell population abundance	Neutrophils are FITC/APC-double-positive.
Gating strategy	Live cells were select by FSC and SSC, and then analyzed by FITC and APC. Neutrophils are FITC/APC-double-positive.

- [x] Tick this box to confirm that a figure exemplifying the gating strategy is provided in the Supplementary Information.
